# An Application of the Social Cognitive Career Theory Model of Career Self-Management to College Athletes’ Career Planning for Life After Sport

**DOI:** 10.3389/fpsyg.2020.00009

**Published:** 2020-01-24

**Authors:** Elodie Wendling, Michael Sagas

**Affiliations:** Department of Sport Management, University of Florida, Gainesville, FL, United States

**Keywords:** student-athletes, career planning for life after sport, Social Cognitive Career Theory, Career Self-Management model, sport career transition

## Abstract

Drawing on Lent and Brown (2013) recently developed Social Cognitive Career Theory (SCCT) model of Career Self-Management (CSM), we aimed to determine the key predictors and underlying theoretical mechanisms of college athletes’ career planning processes for life after sport. Ten variables were operationalized (i.e., career planning for life after sport, career decision self-efficacy, career goals, perceived career planning support from coaches, perceived career planning barriers, conscientiousness, openness, extraversion, neuroticism, and agreeableness) to assess the hypothesized CSM model. A survey design was utilized on a sample of 538 NCAA Division I college athletes in the United States to test the model. The measurement and hypothesized models were tested using Partial Least Squares Structural Equation Modeling (PLS-SEM). The measurement model demonstrated satisfactory reliability and validity for all measures. Several significant direct, indirect, and moderating relationships of the cognitive, contextual, and personality variables on career planning were observed. The CSM model was found to be a useful theoretical framework that explained 62.7% of the variance on career planning. The model, along with the validated measures that support it, can help both researchers and practitioners to leverage facilitating (i.e., self-efficacy, career goals, conscientiousness, openness, and extraversion) and impeding (i.e., career barriers) factors of the career planning processes in their work.

## Introduction

For many student-athletes, their athletic career ends once they have exhausted their athletic eligibility. In 2018, the National Collegiate Athletic Association (NCAA) counted over 480,000 student-athletes and reported that the overwhelming majority of them did not play a sport professionally. In fact, only 1.6% of football players, 0.9% of women’s basketball players, 1.2% of men’s basketball players, 9.5% of baseball players, 6.4% of men’s ice hockey players, and 1.4% of men’s soccer players will move on to compete at the professional level (NCAA, 2018). Although most athletes will leave the competitive sport landscape once they exhaust their eligibility, student-athletes are often not ready to enter the job market upon graduation. The extensive demands of intercollegiate athletics can make it difficult for student-athletes to be prepared for a career after they graduate (Tyrance et al., 2013). Their commitment to sport may leave little time and energy to engage in non-sport-related activities and plan for their vocational future (Martens and Lee, 1998; Wendling et al., 2017).

Given that exploring alternative career options and experiencing non-athletic activities are fundamental steps to the career planning process (McQuown Linnemeyer and Brown, 2010), it may not be unusual for student-athletes to exhibit poor career planning (Adler and Adler, 1987; Baillie and Danish, 1992; Good et al., 1993; Murphy et al., 1996; Martens and Lee, 1998; Lally and Kerr, 2005) and lower levels of career maturity and planning compared to other college students (Brown et al., 2000; Martens and Cox, 2000; McQuown Linnemeyer and Brown, 2010). As a result, student-athletes may experience transition challenges once they leave college sport (Good et al., 1993; Wooten, 2005).

In spite of being aware that their athletic career will inevitably end, college athletes’ intense focus on sport over the years can deter them from exploring viable career options prior to retiring (Pearson and Petitpas, 1990; Murphy et al., 1996; Brown et al., 2000). They may not have enough time during their college years to fully engage in their academics and develop hobbies and interests outside of their sport (Parham, 1993). Thus, student-athletes are likely to postpone major developmental tasks until they are out of college sport, leading to career development deficiencies (Murphy et al., 1996) and a lack of adequate preparation for life after athletics (Tyrance et al., 2013). Given that planning activities prior to retiring were found to reduce the strain associated with the shift in identity and facilitate a transition out of sport (Alfermann et al., 2004; Demulier et al., 2013), it is important to investigate college athletes’ career planning for life after college sport.

As one of the most influential sport career transition models, Taylor and Ogilvie (1994, 2001) Conceptual Model of Adaptation to Career Transition (1994, 2001) highlighted the importance of preretirement planning to facilitate athletes’ adaptation during the transition to life after sport. There has been ample documentation that career planning for life after sport can play a pivotal role in easing transition challenges (Baillie and Danish, 1992; Grove et al., 1997; Alfermann et al., 2004; Lally, 2007; Warriner and Lavallee, 2008; Stambulova et al., 2009; Park et al., 2013). Although career planning is a key predictor of healthy career transitions, there has been little consideration of the theoretical processes underlying career planning, warranting the need to determine the key predictors of planning for a career after sport (Demulier et al., 2013).

To clarify the factors facilitating and impeding career planning, our study’s theoretical basis was derived from the recently developed Social Cognitive Career Theory (SCCT) model of Career Self-Management (CSM) (Lent and Brown, 2013). SCCT has been a valuable theoretical framework to address career concerns, examining career planning and transition of professional athletes (Demulier et al., 2013), career decision-making and planning processes of middle school and high school students (Patton et al., 2004; Rogers et al., 2008; Rogers and Creed, 2011; Zhang et al., 2019), career development of college students (Olson, 2014; Park et al., 2018), and predictions of career choices from various academic majors (Cunningham et al., 2005; Lent et al., 2008).

Building on Bandura’s (1986) social cognitive theory, Lent et al. (1994) developed SCCT, including three interconnected models of career development (i.e., interest development, career choice, and performance). Lent and Brown (2006) added a fourth overlapping model aimed at understanding educational and vocational satisfaction and well-being. In these models, they intended to address specific content-related issues such as identifying factors that foster or hinder the formation of vocational interests and the selection of specific career/academic choices (Lent and Brown, 2013).

Several studies have demonstrated the utility of SCCT in predicting career planning and facilitating transitions and career development (Rogers and Creed, 2000, 2011; Rogers et al., 2008; Creed et al., 2013; Demulier et al., 2013). Career planning, decision-making, job-finding, goal-setting, and negotiating transitions are all considered gradual and developmental career processes that unfold over the life span and are referred to as adaptive career behaviors (Lent and Brown, 2013). In an attempt to respond to the need of investigating processes underlying these behaviors, Lent and Brown (2013) have recently appended a fifth model, named CSM.

Although SCCT’s first four models have received extensive research attention, few studies have tested the recently added CSM model, notably with Lent et al. (2016) examining career exploration and decision-making behaviors among a group of college students. Lim et al. (2016) investigated the developmental task of job search behavior using unemployed job seekers and graduating college senior students, while Tatum et al. (2017) tested the model in the context of workplace sexual identity management. This model is yet to be tested in the context of career planning among college athletes. Responding to both needs of enhancing student-athletes’ career planning (Murphy et al., 1996; Martens and Lee, 1998; Lally and Kerr, 2005; McQuown Linnemeyer and Brown, 2010) and assessing the explanatory utility of this model (Lent and Brown, 2013), the purpose of this study was to determine how the theoretical components (i.e., cognitive, environmental, and personal) of this model are posited to interrelate and jointly operate to influence the career planning process of student-athletes. Given that these components are relatively malleable, examining them can provide practitioners (e.g., career professionals, athletic administrators, coaches, psychologists, sport club officers, among others) and student-athletes with more specific vocational guidance. By forging a theoretical understanding of career planning of an understudied population, we also intend to fill a theoretical and empirical void in the existing literature. This theoretical framework can help both researchers and practitioners uncover facilitating and impeding factors of career planning processes, which could eventually help yield a healthy transition to life after sport. Specifically, we aimed to address each of the following research questions:

1. How are cognitive, contextual, and personality factors posited to interrelate within the CSM model as applied to career planning for life after sport?
2. How much do these predictors contribute to the variance in student-athletes’ career planning, and how do they influence this outcome?

To address these questions, we first introduce the SCCT model of CSM and present the underlying mechanisms associated with career planning before empirically testing these relationships.

### Overview of Social Cognitive Career Theory Model of Career Self-Management

The CSM model was developed to examine how, under varying cognitive, personal, and contextual influences, individuals direct their own career development and navigate career transitions. Changing work environments and unstable economic conditions have made the normative transition from college to work increasingly challenging, requiring college students to acquire adaptable skills and be resilient in the face of adversity (Murphy et al., 2010; Lent, 2013). Given these realities, the emphasis of the CSM model is on the concepts of adaptive career behaviors and personal agency, and how such qualities can help individuals direct their own career development and manage career changes.

First, adaptive career behaviors are related to Savickas (1997) notion of career adaptability, which is defined as “the readiness to cope with the predictable tasks of preparing for and participating in the work role and with the unpredictable adjustments prompted by change in work and working conditions” (p. 254). These behaviors may be employed proactively (e.g., in the context of a normative developmental task such as career planning for life after sport) and reactively (e.g., to cope with challenging career transitions) (Lent and Brown, 2013). Such behaviors refer to processes required for the preparation and adjustment involved in the negotiation of life transitions.

Second, agentic qualities are based on the assumption that individuals have the abilities to “engage in forethought, intentional action, self-reflection, and self-reaction” (Lent and Brown, 2013, p. 558). Being cognizant of the active role they have over adapting to changes can lessen the transition challenges. With these capacities, they can actively and partly direct their own career pursuits in conjunction with environmental influences and resources (Lent and Brown, 2013). Due to the importance of human agency in the CSM model, adaptive career behaviors will thus “enable people to play a part in their self-development, adaption, and self-renewal” (Bandura, 2001, p. 2). Despite instances of factors that are beyond individuals’ control and impede or facilitate career pursuits, personal agency plays a critical part in developing the resilience necessary to alleviate hurdles and minimize challenging career events. Certain personal characteristics and contextual supports may facilitate the exercise of adaptive career behaviors, and in turn these behaviors are deemed instrumental to more distal outcomes such as career transitions (Lent and Brown, 2013). Thus, the CSM model was not proposed to encourage individuals to act alone in directing their career pursuits; instead, the model emphasizes the reciprocal interplay of personal, contextual, and cognitive factors that will influence individuals’ purposive career behaviors (Lent and Brown, 2013).

The CSM model focuses on the dynamic interplay between social cognitive factors, environmental attributes, and personality traits that promote or deter adaptive behaviors, such as the career planning process, the focus of our work. Two key social cognitive variables of SCCT that serve as proximal antecedents of career planning are self-efficacy and goals. Positive interactions between those two central predictors will stimulate and promote career planning (Rogers and Creed, 2011; Demulier et al., 2013). Self-efficacy refers to an individual’s belief of his/her ability to perform a specific task or behavior required to bring forth a desired outcome (Bandura, 1986). In the CSM model, self-efficacy refers to “perceived ability to manage specific tasks necessary for career preparation, entry, adjustment, or change across diverse occupational paths” (Lent and Brown, 2013, p. 561). Goals are defined by Lent et al. (1994) as the intentions to engage in a given behavior in order to achieve a particular outcome. While being influenced by self-efficacy, setting goals helps guide and encourage career planning (Lent et al., 1994; Creed et al., 2013). Indeed, once goals are identified, plans are made to pursue those identified goals, triggering career planning.

Environmental influences, operationalized as career supports and barriers, are critical components of the CSM model as they operate in concert with cognitive variables and provide important practical implications (Lent, 2013). Indeed, individuals may learn to develop plans for coping with these barriers and building on these supports. In this study, supports signify student-athletes’ perceived help, encouragement, and guidance provided by coaches in pursuing their career goals and plans for life after sport. We focused mainly on the supports provided by coaches because they are considered one of the most salient, influential, and/or supportive individuals to student-athletes while in college (Adler and Adler, 1985; Perna et al., 1996; Miller and Kerr, 2002; Kadlcik and Flemr, 2008). Barriers refer to student-athletes’ perceived hurdles that may prevent them from engaging in career planning for life after sport.

Finally, personality consists of a relatively stable set of characteristics that indicate individuals’ tendencies of thinking, acting, and feeling (Brown and Hirschi, 2013). Personality traits are deemed important predictors of career planning given that certain tendencies can facilitate (e.g., conscientiousness, extraversion, openness) or deter (e.g., neuroticism) career planning (Brown and Hirschi, 2013). For instance, conscientiousness (i.e., being planful, self-disciplined, and persevering) was found to help individuals make career plans and to cope with normative transitions (Kadlcik and Flemr, 2008; Rogers et al., 2008; Rogers and Creed, 2011; Demulier et al., 2013). Because cognitive variables affect behavioral outcomes in conjunction with contextual and personality attributes, it is necessary to clarify the impact of each of these factors on career planning (Rogers et al., 2008). In addition, theoretically, contextual and personality factors can serve as moderators of the relationship between career goals and planning (Lent and Brown, 2013).

Overall, cognitive, contextual, and personality inputs interact with each other to affect career planning and distal career transition outcomes. This model was intended to offer predictive mechanisms that identify key predictors shaping individuals’ self-direction in career pursuits (Lent and Brown, 2013). Drawing on the CSM model, we therefore examined the unique and joint contributions of self-efficacy, goals, support and barriers, and personality attributes to the prediction of career planning for life after sport, as well as the underlying relationships among these predictors.

### Current Study

**FIGURE 1:**
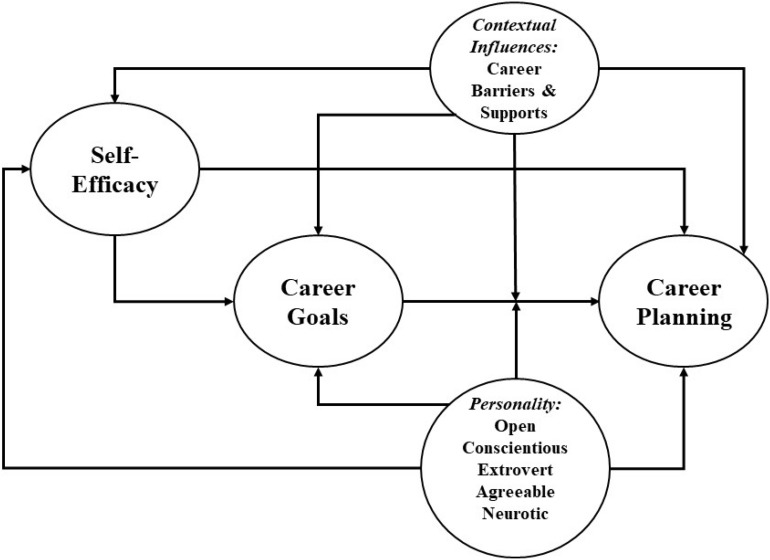
Model of Career Self-Management as applied to career planning behavior. Adapted from Lent and Brown (2013).

The theoretical model we utilized to frame the hypotheses under study is depicted in Figure 1, as adapted by Lent and Brown (2013). Given the complexity of the model and the large number of hypotheses supported by this model, we presented our hypotheses (and results) using a table (Table 1) that summarizes the hypotheses of this study as well as the studies that have shown empirical and conceptual support for these hypotheses, similar to what a study conducted by Rose et al. (2012) did. We first tested direct and indirect relationships of the cognitive variables with career planning through hypotheses 1–4. Contextual variables were then analyzed through hypotheses 5–9, starting with a testing of the direct relations of coaches’ support and career barriers with cognitive variables and career planning (i.e., H5, H6), and followed by a testing of indirect relationships of coaches’ support and career barriers with the cognitive variables and career planning (H7–H9). The direct and indirect relationships of the personality variables with cognitive variables and career planning were analyzed through hypotheses 10–17. Finally, we tested the moderating effect of conscientiousness on the relationship between career goal and career planning (H18). To the best of our knowledge, this moderating effect has not been empirically tested before, and the effect has only been advanced conceptually in the recently developed model of CSM (Lent and Brown, 2013).

**TABLE 1:** Hypotheses and supporting literature.

**Hypotheses**	**Key supporting literature**
**Cognitive variables**	
H1: Self-efficacy is positively related to career goals.	Lent et al. (2008, 2016, 2017), Rogers et al. (2008), Creed et al. (2013), Lent and Brown (2013)*, Lim et al. (2016), Roche et al. (2017).
H2: Career goals is positively related to career planning.	Rogers et al. (2008), Rogers and Creed (2000, 2011)*, Creed et al. (2013), Demulier et al. (2013), Lent and Brown (2013)*, Lim et al. (2016).
H3: Self-efficacy is positively related to career planning.	Rogers et al. (2008), Rogers and Creed (2011), Demulier et al. (2013), Lent and Brown (2013)*, Renn et al. (2014), Lent et al. (2016).
H4: Career goals partially mediates the relationship between self-efficacy and career planning.	Lent and Brown (2013)*, Lim et al. (2016).
**Contextual variables**	
H5: Coaches support is positively related to self-efficacy, career goals, and career planning.	Lent et al. (2008, 2016), Rogers et al. (2008), Rogers and Creed (2011), Lent and Brown (2013)*, Renn et al. (2014), Lim et al. (2016).
H6: Barriers are negatively related to self-efficacy, career goals, and career planning.	Lent et al. (2008, 2016), Lent and Brown (2013)*.
H7: Self-efficacy partially mediates the relationship between coaches support/career barriers and career goals.	Bandura (2000)*, Lent et al. (2008, 2016), Lent and Brown (2013)*, Lim et al. (2016).
H8: Self-efficacy partially mediates the relationship between coaches support/career barriers and career planning.	Lent and Brown (2013)*, Renn et al. (2014), Lim et al. (2016).
H9: Career goals partially mediates the relationship between coaches support/career barriers and career planning.	Lent and Brown (2013)*, Lim et al. (2016).
**Personality variables**	
H10: Conscientiousness and extraversion are positively related to self-efficacy.	Wang et al. (2006), Hartman and Betz (2007), Rogers et al. (2008), Demulier et al. (2013), Lent et al. (2016), Lim et al. (2016), Roche et al. (2017).
H11: Neuroticism is negatively related to self-efficacy.	Wang et al. (2006), Hartman and Betz (2007), Rogers et al. (2008), Brown and Hirschi (2013)*.
H12: Conscientiousness, extraversion, and openness are positively related to career goals.	Brown and Hirschi (2013)*, Demulier et al. (2013), Lent and Brown (2013)*, Lent et al. (2016), Lim et al. (2016).
H13: Conscientiousness, extraversion, and openness are positively related to career planning.	Rogers et al. (2008), Rogers and Creed (2011), Demulier et al. (2013), Lent and Brown (2013)*, Lent et al. (2016).
H14: Neuroticism and agreeableness are negatively related to career planning.	Hirschi et al. (2011), Rogers and Creed (2011).
H15: Self-efficacy partially mediates the relationship between conscientiousness and career goals.	Lent et al. (2016), Lim et al. (2016), Roche et al. (2017).
H16: Self-efficacy partially mediates the relationship between conscientiousness, openness, and career planning.	Rogers et al. (2008), Demulier et al. (2013), Lim et al. (2016).
H17: Career goals partially mediate the relationship between conscientiousness, openness, and career planning.	Rogers et al. (2008), Demulier et al. (2013), Lim et al. (2016).
H18: The relationship between career goals and career planning is moderated by conscientiousness, such that higher levels of conscientiousness lead to a stronger relationship of career goals to career planning.	Lent and Brown (2013)*

*Conceptual article.

## Materials and Methods

### Sample Design and Data Collection

In this study, we used a cross-sectional survey design. The target population consisted of all NCAA Division I student-athletes in the United States. The Division I represents the highest level of competition in the college sport system in the United States. Approximately, 180,000 student-athletes participate in this division and around 60% of them are on an athletic scholarship (NCAA, 2019). All NCAA Division I institutions (i.e., 350 colleges and universities) were invited to participate in this study. We contacted key athletic administrators at each of these institutions to request their assistance in disseminating an online questionnaire (hosted by Qualtrics) to the student-athletes at their school. In total, 21 universities located in 15 different states volunteered to participate and distribute the online questionnaire to their group of student-athletes. An incentive was offered to participants such that every 20th participant that completed the questionnaire were awarded a $25 Amazon gift card. Participants were informed that their participation was anonymous and voluntary, and provided their consent to participate in the study by completing the questionnaire.

A total of 1,020 student-athletes started the survey, but only 684 fully completed it. Participants who finished the survey in an unrealistically short amount of time or selected the same response for every question were deleted in the data cleaning process, resulting in a total of 538 questionnaires that were retained for data analysis. Thus, ∼53% of the student-athletes who started the survey completed all sections of the protocol. Table 2 provides information about demographics of the participants. A total of 73% (*n* = 393) were female and 27% (*n* = 145) were male, and the age range was 18–23 years, with a mean and median of 20 years old. The sample was comprised of 27% (*n* = 145) freshmen, 21% (*n* = 113) sophomores, 23% (*n* = 124) juniors, 26% (*n* = 140) seniors, and 3% (*n* = 16) graduate students. The racial composition was 80% (*n* = 430) Whites, 7% (*n* = 38) African Americans, 6% (*n* = 32) Hispanics, 4% (*n* = 22) Asians, 2% (*n* = 11) Other, and 1% (*n* = 5) preferred not to respond. A total of 16% (*n* = 86) of our participants were first generation college (FGC) students. A total of 49% of the respondents participated in individual sports (e.g., tennis and golf), while 51% of them participated in team sports (e.g., baseball and basketball). Finally, 33% (*n* = 178) of our group of student-athletes reported that they have already visited the Career Services office on their campus. Our dataset contained 13 missing values, of which 8 were missing on one of the indicators (or 1.5% of missing values in this indicator); thereby, mean replacement was deemed appropriate to use to replace missing values (Hair et al., 2017).

**TABLE 2:** Participants’ demographics.

**Demographic Characteristic**		**Percentage (%)**
Gender	Male	73
	Female	27
Ethnicity	White	80
	African-American	6
	Hispanic	4
	Asian	2
	Other	1
Academic class	Freshman	27
	Sophomore	21
	Junior	23
	Senior	26
	Graduate student	3
Sport type	Individual sport	49
	Team sport	51

### Research Instrument

Lent and Brown (2013) encouraged researchers to investigate the application of various developmental tasks such as the one central to our study (i.e., career planning) using the set of predictors aligned in the CSM model. However, it was clear that the operationalization of these variables needed to be altered to establish a valid application of the CSM model. The 10 variables used to assess the hypothesized CSM model were operationalized using new and existing scales that were modified and adapted to our specific context, as recommended by Lent and Brown (2013).

The items selected for the questionnaire followed established scale development procedures (DeVellis, 2003). An extensive literature review was conducted to generate items that reflect the theoretical definition of each construct and accurately capture the intended meaning of the variables in question. Follow up discussions were ensued with three experts of the domain of interest and measurement to ensure face and content validity of the items. Grammatical and structural changes were made to improve clarity and the readability of the survey.

Based on the above procedures, scale items used for 7 of the 10 constructs in this study were adapted from existing scales that have shown acceptable reliability and validity in previous studies. *Self-efficacy* was measured with 18 items adapted from the 25-item Career Decision Self-Efficacy-Short Form scale (CDSE-SF; Betz et al., 1996). *Coaches’ support* was operationalized using seven items adapted from the Parents’ Influence factor of the Career Influence Inventory (CII; Fisher and Stafford, 1999). Finally, we used the short 15-item Big Five Inventory (BFI-S; Gerlitz and Schupp, 2005) to measure the five personality factors (i.e., *openness*, *conscientiousness*, *extraversion*, *agreeableness*, and *neuroticism*). For each of these five factors, we added one item selected from the 44-item Big Five Inventory (BFI; John and Srivastava, 1999). Thus, 20 items in total were used to assess the personality dimensions.

New scales were developed for three constructs, given that existing scales either needed to be improved in terms of validity (i.e., career goals) or were not deemed appropriate for our context (i.e., perceived career barriers and career planning for life after sport). The *career goals* variable assesses the extent to which student-athletes have set career goals that they intend to pursue in order to achieve their career plans. For this variable, we adapted the first item from Mu (1998) 6-item scale of career goal setting and created five other items. The *perceived career barriers* variable measures the extent to which student-athletes perceive hurdles that may prevent them from planning a career for life after sport. For this variable, we created 11 items that were barriers related to making career plans, and nine items were developed using the revised Perceptions of Educational Barriers scale (PEB-R; McWhirter, 2000).

Viewed as an ongoing and life-long activity commonly used during life transitions (Creed et al., 2013), the *career planning* variable measures the extent to which student-athletes established career plans and prepared for a career after sport. We measured career planning for life after sport adapting three items from existing scales: “I’m having difficulties preparing myself for a career after college sport” was modified from the Counseling Form of the Career Maturity Inventory (CMI-C; Savickas and Porfeli, 2011), and “I have formulated a viable plan for my career after college sport” and “I have determined a specific plan to gain practical experience in the field I plan to pursue after college” were adapted from the career planning subtask of the Student Developmental Task and Lifestyle Inventory (SDTLI; Winston et al., 1987). We also created five items, which were as follows: “I have a good understanding of the steps I need to take to pursue my career plans,” “I am unsure about what my career plans for life after sport should be yet,” “I have gathered detailed information about career requirements, employment trends, and ways of getting into occupations that interest me,” “I’m taking the steps necessary to pursue my career plans,” and “I am too busy at this point to make career plans for life after college.”

We pre-tested these three variables through a pilot test with 51 student-athletes (that were not participants of the main study), resulting in several items being further refined. Initial results demonstrated acceptable reliability estimates (α > 0.7). All questionnaire items were reflective and except for self-efficacy, rated on a seven-point Likert type scale ranging from 1 (strongly disagree) to 7 (strongly agree). Self-efficacy was measured on a seven-point Likert type scale, anchored with 1 = no confidence at all and 7 = complete confidence.

### Data Analysis

Measurement and hypothesized models were tested using Partial Least Squares Structural Equation Modeling (PLS-SEM) in the SmartPLS (version 3.2.7) software. Maximizing the explained variance of endogenous latent variables, PLS is a well-established analytical method that is appropriate for research aims that are focused on predictions and theory building (Hair et al., 2017). Although SCCT is a well-established theory, the CSM model has recently been developed, and some of its relationships have not been tested yet. PLS-SEM has been utilized in the psychology literature (e.g., Westman et al., 2017), and also in career studies (e.g., Waters, 2004; Hsieh and Huang, 2014; Ren and Chadee, 2017). Its use is also recommended for estimating complex models that have many latent variables and indicators and test mediating effects as well as continuous moderator influences (Hair et al., 2017), as is the case here. Another advantage of this technique is that no assumptions about the data distribution are made to estimate model parameters.

A drawback to this approach, relative to its covariance-based SEM sibling, concerns the notion of fit that remains in early stages of development, making the identification of model misspecifications and theory confirmation more challenging to undertake. However, PLS-SEM was designed for prediction rather than explanatory purposes; thereby, the focus is on assessing how well models predict endogenous variables, fostering theory development (Hair et al., 2017). Goodness of fit measures are of little interest with such a purpose; instead, key criteria used to assess the adequacy of the model include the significance of path coefficients, the *f*^2^ effect size, the predictive accuracy as shown by *R*^2^-values, and the predictive relevance as measured by the *Q*^2^-values. We assessed the model using a two-stage analytical procedure for PLS-SEM as delineated by Hair et al. (2017): (1) We tested the measurement model to refine our measures and establish validity and reliability; (2) We examined the structural model and tested the significance of path coefficients using a bootstrapping method with 5,000 resamples.

## Results

### Measurement Model

Items for which the loading exceeded the recommended value of 0.6 (Chin et al., 2008) were retained in the analysis. Decisions of items deletion were based on both empirical analyses and conceptual underpinnings. Supplementary Appendix A provides all items retained and used in this study as well as their sources. Descriptive statistics and psychometric properties of the constructs are presented in Table 3. The internal consistency of the measures was deemed satisfactory given that the composite reliability values all exceeded the recommended value of 0.7, and none of them exceeded 0.95 (Hair et al., 2017). Values >0.95 are not necessarily desirable for validity purposes, as such a high reliability may signify that the indicators measuring the latent variables are too similar and redundant (Hair et al., 2017). The convergent validity was assessed through the average variance extracted (AVE) values. All AVE values exceeded the recommended threshold of 0.5 (Fornell and Larcker, 1981), which indicates that the latent variables in our model explained on average >50% of the variance of their corresponding indicators.

**TABLE 3:** Descriptive statistics and psychometric properties of the constructs.

**Constructs**	**Number of items**	***M***	***SD***	**Loadings range**	**CR**	**AVE**
1. Self-efficacy	12	5.089	0.947	0.663–0.820	0.939	0.564
2. Career goals	5	5.520	1.095	0.684–0.881	0.899	0.644
3. Career planning	8	4.846	1.126	0.621–0.869	0.909	0.560
4. Coaches support	7	5.414	1.213	0.735–0.857	0.931	0.660
5. Career barriers	10	3.166	1.267	0.647–0.797	0.921	0.540
6. Conscientiousness	4	5.724	0.780	0.614–0.791	0.829	0.551
7. Extraversion	4	5.003	1.023	0.692–0.867	0.882	0.653
8. Openness	4	5.300	0.982	0.610–0.848	0.842	0.576
9. Neuroticism	4	3.663	1.093	0.522–0.888	0.804	0.520
10. Agreeableness	4	5.654	0.882	0.589–0.881	0.796	0.501

*Scale reliability: CR > 0.70; convergent validity: AVE > 0.50 and loadings >0.5.*

To establish discriminant validity, we used the Fornell–Larcker criterion analyses, which requires that the square root of each variable’s AVE values be greater than the highest correlation between the variable in question and all other latent variables in the model. Table 4 presents the square root of AVE in bold and placed on the diagonal while the bivariate correlations of all 10 latent variables are shown on the off-diagonal in the correlation matrix. This analysis demonstrated adequate discriminant validity given that each latent variable shared more variance with its related indicators than with any other constructs. However, Henseler et al. (2015) have recently scrutinized this approach and found some issues in terms of detecting a lack of discriminant validity. As an alternative approach, they recommended to use the heterotrait-monotrait (HTMT) ratio of correlations. Results of this analysis are presented in Table 5. All HTMT values were below the conservative threshold of 0.85 (Henseler et al., 2015), and the bootstrap confidence interval results of HTMT statistic did not include the value of 1 for all combinations of constructs, showing that each construct in this study is empirically distinct. Therefore, we demonstrated satisfactory reliability and validity of our measurement model.

**TABLE 4:** Fornell–Larcker criterion analyses for discriminant validity and correlation matrix.

**Constructs**	**1**	**2**	**3**	**4**	**5**	**6**	**7**	**8**	**9**	**10**
1. Self-efficacy	**0.751**									
2. Career goals	0.602**	**0.802**								
3. Career planning	0.627**	0.738**	**0.748**							
4. Coaches support	0.165**	0.181**	0.136**	**0.812**						
5. Career barriers	−0.475**	−0.419**	−0.489**	−0.156**	**0.735**					
6. Conscientiousness	0.297**	0.378**	0.333**	0.151**	−0.299**	**0.742**				
7. Extraversion	0.204**	0.119**	0.138**	0.081	−0.185**	0.110*	**0.808**			
8. Openness	0.191**	0.150**	0.109*	0.052	–0.043	0.276**	0.280**	**0.759**		
9. Neuroticism	−0.209**	−0.145**	−0.136**	−0.115**	0.209**	−0.270**	−0.097*	−0.255**	**0.721**	
10. Agreeableness	0.149**	0.179**	0.134**	0.140**	−0.105*	0.449**	0.040	0.208**	−0.248**	**0**.**707**

Bold-faced numerals on the diagonal represent the square root of the average variance extracted while the off-diagonal values are correlations. Discriminant validity: square root of the AVE values are all greater than correlation coefficients. *p < 0.05; **p < 0.01.

**TABLE 5:** Heterotrait-monotrait (HTMT) analysis.

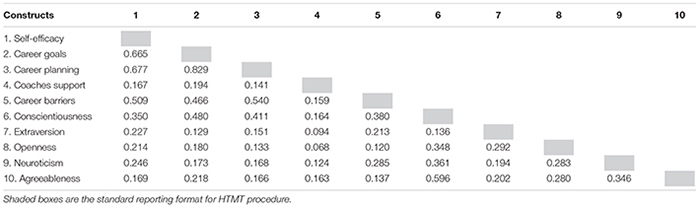

### Structural Model

Before assessing the structural model and its predictive power and relevance, we checked for collinearity issues by examining the variance inflation factor (VIF) values of all sets of predictor constructs in the structural model. No collinearity issues were found, as all VIF values were well below the threshold value of five (Hair et al., 2017). We included four control variables in the model (i.e., age, gender, race, and FGC students), given that previous work have controlled for their effects and have shown their influence on our outcomes of interest (Patton and Creed, 2001; Patton et al., 2004; Creed et al., 2007; Rogers et al., 2008; Vuong et al., 2010; Rogers and Creed, 2011; Olson, 2014). The age range of our participants was 18–23 years old. Gender was coded as 0 for male and 1 for female, and FGC students were coded as 0 for no and 1 for yes. Age was found to be significantly related to self-efficacy (β = 0.083; *p* < 0.05) and career goals (β = 0.088; *p* < 0.05). Gender was found to be significantly related to self-efficacy (β = −0.109; *p* < 0.01), showing that our male participants had more confidence in making career decisions than their female counterparts. Finally, no significant relationships were found for race and FGC students.

The bootstrapping method of 5,000 iterations provided the statistical significance of the proposed direct, indirect, and moderating effects. All path estimates of the hypothesized model and their significance are reported in Table 6. For clarity of the summary of hypothesis testing, we included the conclusion drawn for each of them from the statistical findings in Table 6. For the direct and moderating effects, we also reported and assessed the relevance of significant relationships using *f*^2^ as recommended by Hair et al. (2017). Indeed, some path coefficients can be significant but also have a very small effect, which would not warrant much practical attention.

**TABLE 6:** Structural model path estimates and hypotheses testing.

**Path of research model**	**Beta**	***t*-Value**	***F*-Square**	**Hypothesis decision**
**Cognitive variables**				
SE → Goals	0.482	12.443***	0.290	Hypothesis 1 is supported.
Goals → Plan	0.527	15.795***	0.418	Hypothesis 2 is supported.
SE → Plan	0.240	6.111***	0.084	Hypothesis 3 is supported.
SE → Goals → Plan	0.254	9.759***		Hypothesis 4 is supported; partial mediating effect of goals.
**Contextual variables**				
CS → SE	0.066	1.627	0.006	Hypothesis 5 is not supported.
CS → Goals	0.057	1.579	0.005	Hypothesis 5 is not supported.
CS → Plan	–0.023	0.830	0.001	Hypothesis 5 is not supported.
Barriers → SE	–0.401	10.060***	0.190	Hypothesis 6 is supported.
Barriers → Goals	–0.137	3.555***	0.023	Hypothesis 6 is supported.
Barriers → Plan	–0.152	4.369***	0.042	Hypothesis 6 is supported.
Barriers → SE → Goals	–0.193	7.903***		Hypothesis 7 is partially supported; no partial mediating effect of SE on CS and goals.
CS → SE → Goals	0.032	1.576		Hypothesis 7 is partially supported; partial mediating effect of SE on barriers and goals only.
Barriers → SE → Plan	–0.096	5.126***		Hypothesis 8 is partially supported; no partial mediating effect of SE on CS and plan.
CS → SE → Plan	0.016	1.566		Hypothesis 8 is partially supported; partial mediating effect of SE on barriers and plan only.
Barriers → Goals → Plan	–0.072	3.540***		Hypothesis 9 partially supported; no partial mediating effect of goals on CS and plan.
CS → Goals → Plan	0.030	1.552		Hypothesis 9 partially supported; partial mediating effect of goals on barriers and plan only.
**Personality variables**				
Consc → SE	0.111	2.540**	0.012	Hypothesis 10 is supported.
Extra → SE	0.078	2.924*	0.007	Hypothesis 10 is supported.
Neuro → SE	–0.051	1.329	0.003	Hypothesis 11 is not supported.
Consc → Goals	0.195	4.560***	0.045	Hypothesis 12 is partially supported; Extra and Goals are not related to goals.
Extra → Goals	–0.030	0.860	0.001	Hypothesis 12 is partially supported; only being conscientious is related to goals.
Open → Goals	0.015	0.402	0.000	Hypothesis 12 is partially supported; only being conscientious is related to goals.
Consc → Planning	0.039	1.175	0.003	Hypothesis 13 is not supported.
Extra → Planning	0.006	0.181	0.000	Hypothesis 13 is not supported.
Open → Planning	–0.026	0.858	0.001	Hypothesis 13 is not supported.
Neuro → Planning	0.020	0.681	0.001	Hypothesis 14 is not supported.
Agree → Planning	–0.016	0.483	0.001	Hypothesis 14 is not supported.
Consc → SE → Goals	0.053	2.488**		Hypothesis 15 is supported; partial mediating effect of SE on consc and goals.
Consc → SE → Planning	0.027	2.294*		Hypothesis 16 is partially supported; full mediating effect of SE rather than partial.
Open → SE → Planning	0.025	2.281*		Hypothesis 16 is partially supported; full mediating effect of SE rather than partial.
Consc → Goals → Planning	0.103	4.328***		Hypothesis 17 is partially supported; full mediating effect of goals rather than partial.
Open → Goals → Planning	0.008	0.401		Hypothesis 17 is partially supported; goals fully mediates only conscientious and not open.
Consc * Goals → Planning	0.097	3.541***	0.026	Hypothesis 18 is supported.

SE, self-efficacy; CS, coaches support; Goals, career goals; Plan, career planning; Consc, conscientious; Extra, extravert; Agree, agreeable, Neuro, neurotic. Critical *t*-values *1.96 (*p* < 0.05); **2.58 (*p* < 0.01); ***3.29 (*p* < 0.001).

#### Direct Path Analysis

To assess the effect size of the direct relationships, Cohen (1988) guidelines were used, in which 0.02 indicated small effects, 0.15 medium effects, and 0.35 large effects. The relationships between self-efficacy and career goals (H1) (β = 0.482; *p* < 0.001), and career goals and planning (H2) (β = 0.527; *p* < 0.001) were found to have a large effect size. An effect size in between medium and large effects was observed for the relationship between career barriers and self-efficacy (H6) (β = −0.401; *p* < 0.001). The relationship between self-efficacy and career planning (H3) (β = 0.240; *p* < 0.001) had an effect size between small and medium effects. Small effects were found for the relationships between career barriers and goals (H6) (β = −0.137; *p* < 0.001), career barriers and planning (H6) (β = −0.152; *p* < 0.001), and conscientiousness and career goals (H12) (β = 0.195; *p* < 0.001). Finally, significant positive relationships were found between conscientiousness and self-efficacy (H10) (β = 0.111; *p* < 0.01), extraversion and self-efficacy (H10) (β = 0.078; *p* < 0.05). An additional direct and significant relationship that has not been previously tested in the literature was found between openness and self-efficacy (β = 0.103; *p* < 0.05). Overall, these results provided support for H1, H2, H3, H6, and H10. Partial support was found for H12 given that only conscientiousness was related to career goals while openness and extraversion were not. No significant relationships were found for coaches’ support with all three cognitive variables, and none of the personality factors was related to career planning.

#### Mediation Analysis

We used the mediation analysis procedure recommended by Hair et al. (2017). If the path including the mediator variable under test (i.e., the indirect effect) is significant, we then need to determine the statistical significance of the direct effect. Partial mediation is found if the direct link is significant, whereas support for full mediation is demonstrated if the direct link is not significant. Among the mediating effects tested in the model, we found that career goals acted as a partial mediator of the relationships between self-efficacy and career planning (H4) (β = 0.254; *p* < 0.001), and barriers and planning (H9) (β = −0.072; *p* < 0.001). Self-efficacy also acted as a partial mediator between barriers and goals (H7) (β = −0.193; *p* < 0.001), career barriers and planning (H8) (β = −0.096; *p* < 0.001), and conscientiousness and goals (H15) (β = 0.053; *p* < 0.01). All partial mediations were complementary given the indirect and direct effects pointed in the same direction. We did not anticipate that the effects of conscientiousness on career planning would be fully mediated by self-efficacy (H16) (β = 0.027; *p* < 0.05) and by goals (H17) (β = 0.103; *p* < 0.001). Self-efficacy also fully mediated the effect of openness on planning (H16) (β = 0.025; *p* < 0.05).

An indirect and significant relationship that has not been previously tested in the literature was found between openness and career goals via self-efficacy (β = 0.049; *p* < 0.05). In this case, full mediation was supported as openness was not directly related to goals, only indirectly through self-efficacy. Overall, the results of the indirect relationships provided support for H4 and H15. Partial support was found for H7, H8, and H9 since only barriers were indirectly related to career goals and planning, while no indirect links were found for coaches’ support. Finally, H16 and H17 were partially supported given that we found full rather than partial mediations of self-efficacy on conscientiousness and planning, and on openness and planning, as well as full mediation of career goals on conscientiousness and planning. These results mean that conscientiousness and openness were not directly related to career planning, only indirectly via self-efficacy and goals.

#### Moderation Analysis

We followed the two-stage approach proposed by Chin et al. (2003), which is appropriate to test the significance of moderating effects. A significant and positive interaction effect of conscientiousness on the relationship between career goals and planning was obtained (H18) (β = 0.097; *p* < 0.001). The interaction term’s effect size indicated a large effect with *f*^2^ value of 0.026, according to Kenny (2016) guidelines (i.e., 0.005 indicated small effects, 0.01 medium effects, and 0.025 large effects). This result means that higher levels of conscientiousness strengthen the relationship between career goals and planning, while lower levels of conscientiousness weaken the relationship between career goals and planning.

**FIGURE 2:**
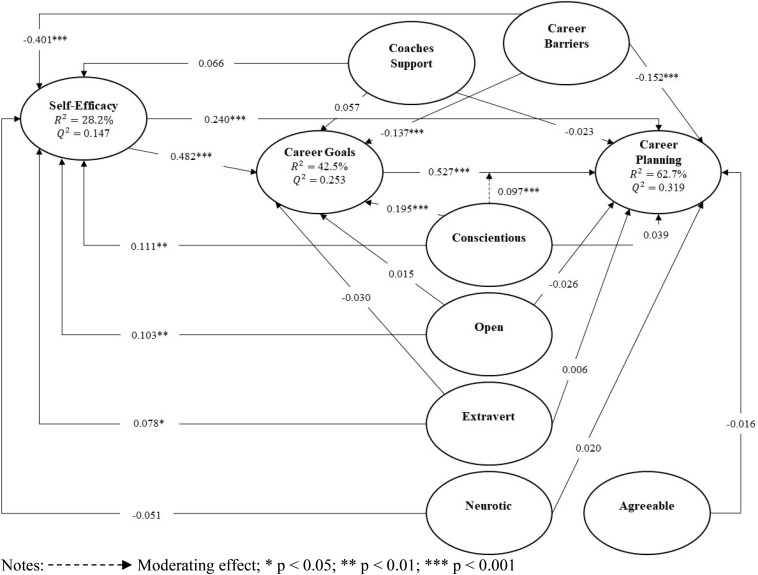
Structural model.

Results of the research model are summarized in Figure 2, including all hypothesized path coefficients and their significance. The predictive accuracy (*R*^2^) and relevance (*Q*^2^) of the predictors on the endogenous variables were also indicated and demonstrated very good predictive power and relevance. The *R*^2^ for career planning is rather substantial with 62.7% of the variance in career planning being explained by all the other variables in the model. Furthermore, a moderate predictive power was found for career goals, with 42.5% of the variance in career goals being explained by all the other variables in the model except planning. Finally, 28.2% of the variance in self-efficacy was explained by all the other variables in the model excluding goals and planning.

In order to assess the predictive relevance, the Stone–Geisser’s *Q*^2^-values for endogenous variables (Geisser, 1974; Stone, 1974) were also calculated using the blindfolding procedure for an omission distance of 7. A value larger than zero suggests that the path model exhibits predictive relevance for a particular endogenous variable, which means that “it accurately predicts data not used in the model estimation” (Hair et al., 2017, p. 202). If a *Q*^2^-value is smaller than zero, the model lacks predictive relevance. Results yielded *Q*^2^-values of 0.147 for self-efficacy, 0.253 for career goals, and 0.319 for career planning, which are all substantially above the threshold value of 0, demonstrating acceptable predictive relevance for all three endogenous latent variables.

## Discussion

The main purpose of this study was to determine the key antecedents and underlying theoretical mechanisms of student-athletes’ career planning processes for life after sport. Before testing hypothesized relationships, we demonstrated satisfactory internal consistency reliability, convergent validity, and discriminant validity of our measurement model. In addition to providing initial support for the psychometric properties of the measures used in this study, we also observed strong predictive adequacy of the CSM model as applied to career planning for life after sport. Among the direct predictors of career planning, we found that self-efficacy and career goals acted as facilitators whereas perceived barriers acted as hindrances of such a process, which is consistent with previous studies (Creed et al., 2013; Demulier et al., 2013; Lent and Brown, 2013). Evidencing further support for the CSM model, self-efficacy, career goals, and career planning demonstrated significant positive interactions with each other, which suggest that setting career goals and planning for a career after sport are facilitated by enhanced confidence in making career decisions. Given that previous research demonstrated that higher self-efficacy led to enhanced behavioral competence (Choi et al., 2012), self-efficacy plays a critical role in fostering the formation and elaboration of goals, which in turn stimulates and guides career planning processes (Rogers and Creed, 2000, 2011). Indeed, student-athletes with higher self-efficacy will regard such activities as important tasks to master rather than challenges to avoid.

Although student-athletes in this study did not seem to perceive a large number of career barriers, those who did, reported lower scores on self-efficacy, career goals, and planning. In addition, career barriers negatively affected career goals through a decreased confidence in making career decisions, as was the case in previous work (Lent et al., 2008). Further empirical evidence was observed such that the presence of career barriers indirectly deterred student-athletes to plan for a career after sport either via a decrease in self-efficacy or via a lack of setting career goals. Therefore, student-athletes who fail to engage in career planning for life after sport may lack confidence in making career decisions, have difficulties in articulating clear and specific career goals, and may be affected by career barriers.

Unexpectedly, career planning for life after sport did not directly depend on a number of interrelated predictors, including perceived support from coaches and all five personality factors. Given that coaches spend a large amount of time with college athletes and can exert control over athletes’ decisions, we would have expected coaches to have a salient influence over their athletes’ career choices and planning. Although coaches were perceived by our group of athletes as highly supportive toward their career plans, they did not have much of an impact on those plans. Indeed, career support from coaches did not have any direct and indirect influences on any of the three core variables of the model. This discrepancy with previous work may come from the various ways supports have been measured. Social support has been assessed either specifically by designating a social role such as mentors, parents, teachers, and friends or broadly without referring to a specific supporter. This lack of consistency in measuring supports can explain contradictory results. For instance, Rogers et al. (2008) reported a measure of career support from parents, teachers, and friends, and observed a direct influence on career planning, as well as a positive moderating effect of support on career goals and planning. On the contrary, measuring social support in general, Lim et al. (2016) found no direct links of supports with goals and actions as well as no moderating effects of supports on goals and actions, which were consistent with our findings.

Regarding the personality influences, only a few career studies have tested the relationships of all five personality factors with SCCT variables (viz., Rogers et al., 2008; Rogers and Creed, 2011), as past research mainly used conscientiousness (e.g., Demulier et al., 2013; Lent et al., 2016; Lim et al., 2016). Conscientiousness, openness, and extraversion were the only personality traits that had important impacts on the three core variables of the model. Contrary to previous findings (Rogers et al., 2008; Hirschi et al., 2011; Rogers and Creed, 2011), being emotionally unstable and having negative feelings (i.e., neuroticism) and being loyal and cooperative (i.e., agreeableness) (Costa and McCrae, 1992) had no influences on the three core variables of the model.

Student-athletes who were conscientious, extroverted, or open were more confident in their ability to make career decisions while only the quality of being conscientious positively affected career goals, findings that were in line with previous work (Rogers et al., 2008; Demulier et al., 2013; Lent et al., 2016; Lim et al., 2016). Although extraversion and openness did not directly affect career goals and planning, both traits indirectly facilitated student-athletes’ career goals and planning via enhanced self-efficacy. Furthermore, conscientiousness was of particular interest in this study given individuals’ tendencies to be organized, responsible, perseverant, accountable, and planful (Costa and McCrae, 1992). Unexpectedly, this trait was not directly related to career planning but only indirectly through the mediating effects of both self-efficacy and career goals, and through its positive moderating effect on the direct link between goals and planning. Although the lack of direct influences from conscientiousness may be contrary to previous studies (Rogers et al., 2008; Rogers and Creed, 2011; Demulier et al., 2013), the positive indirect and moderating effects of this trait on career planning still qualify it as one of the most important traits to possess to facilitate adaptation and in turn anticipate a healthier transition to life after sport. Indeed, being conscientious enhances career decision self-efficacy and encourages career goal-setting, which in turn facilitates career planning.

Although it was conceptually advanced that personality traits and contextual influences were posited to moderate the relationship between career goals and planning in the CSM model (Lent et al., 2000; Lent and Brown, 2013), empirical evidence is still lagging behind. In our study, we found that the tendency of being conscientious strengthened the relationship between career goals and career planning, making it an important attribute for student-athletes to develop in order to ensure that their goals are translated into plans. Along with providing empirical evidence of previously untested links in the CSM model, we extended the domain of the theory by testing the entire CSM model to a group of elite college athletes and demonstrating its applicability to this understudied population. We were also able to improve our understanding of the career planning process by examining the role of key determinants that shape such processes. Therefore, we shed light on the vocational guidance needed to prepare student-athletes for a career once they leave college sport and highlighted the value of developing plans for a career prior to graduating from college.

### Limitations, Future Research, and Implications for Practice

The primary limitations found in the study concerned the sampling procedure and the cross-sectional nature of the data collection. Given the difficulty to reach NCAA college athletes, we used a non-probability sampling procedure. Furthermore, our sample was overrepresented by female student-athletes. Thus, our sample of NCAA Division I student-athletes may not be fully representative of the target population, limiting our ability to generalize the conclusions found in this study to our entire population of interest. In addition, cross-sectional designs limit the causality in the hypothesized relationships by only examining the relations among the variables; hence, we cannot assert that the antecedents are causally related to desired outcomes. As a result, future research could further test the full temporal sequence proposed in the CSM model using a longitudinal design or quasi-experiment. We also encourage additional inquiry to further scrutinize psychometric properties of the measures used in this study and theoretical predictions of this model. Furthermore, self-report measures alone cannot possibly capture a thorough and detailed understanding of such an idiosyncratic experience as the career planning process. Thus, the present results can be complemented with qualitative inquiries conducted on a group of athletes in transition to shed light on the complexities of this career planning process and its impact on the transition to life after sport.

Given that the college years are an important developmental period for many emerging adults to shape their identity for adulthood and make career decisions (Arnett, 2007), it is critical to provide student-athletes with adequate learning opportunities that could encourage them to plan for a career after sport. Given that the CSM model explained 62.7% of the variance in career planning, we demonstrated that this theoretical framework can help inform educational and preventive programming specifically designed to enhance student-athletes’ career planning skills. Career planning courses offered to student-athletes could therefore be assessed using the measures and model provided in this study to examine their effectiveness. The practical utility of this model suggests that career interventions can focus on addressing cognitive, contextual, and personal influences to acquire the resilience necessary to plan for a career after sport. In addition to developing key personal tendencies such as conscientiousness, openness, and extraversion, proactive career management strategies including efficacy-enhancing, goals-setting, and barrier-coping can either directly or indirectly affect student-athletes’ planning for a career after college sport.

Career professionals, athletic administrators, coaches, sport psychologists, and parents can help strengthen student-athletes’ self-efficacy in making career decisions, which in turn, would encourage them to set and pursue realistic career goals. Those goals would be more likely translated into career plans with student-athletes taking the necessary steps to make progress toward identified goals. They can also help them cultivate a support system and prepare strategies to cope with anticipated barriers. Although proactively managing contextual factors and anticipating adverse career events may not always be feasible, student-athletes must be able to remain vigilant and be prepared to respond to potential setbacks and difficulties in pursuing their career plans.

Given that conscientiousness, openness, and extraversion tended to facilitate career planning processes, administrators, coaches, and other constituents can help athletes who score low on those traits to recognize the value of these attributes in managing their own career behaviors. Personality tendencies are partly dispositional traits but also malleable enough to be developed and acquired through training sessions (Lent and Brown, 2013). Thus, practitioners may encourage student-athletes to develop these characteristics through cognitive and emotional developmental training programs, behavioral modeling, and counseling.

## Conclusion

The CSM model was found to be a useful theoretical framework to predict career planning. This study intended to address an empirical gap in the literature by providing an in-depth understanding of the career planning process among college athletes. We also sought to test and extend the domain of the theory by demonstrating the predictive utility of the CSM model to student-athletes’ career planning for life after sport. The comprehensive model we tested was shown to be well-suited to prescribe solutions to enhance student-athletes’ career planning. Therefore, we contend that this theoretical framework can help both researchers and practitioners uncover facilitating and impeding factors of career planning processes.

## Data Availability Statement

The datasets generated for this study are available on request to the corresponding author.

## Ethics Statement

The studies involving human participants were reviewed and approved by the University of Florida IRB. The patients/participants provided their informed consent to participate in this study by completing the questionnaire.

## Author Contributions

EW and MS contributed to the conception and design of the study. EW conducted the statistical analyses and wrote the first draft of the manuscript. MS wrote several sections of the manuscript. Both authors contributed to the manuscript revision, and read and approved the submitted version of the manuscript.

## Conflict of Interest

The authors declare that the research was conducted in the absence of any commercial or financial relationships that could be construed as a potential conflict of interest.
