# Supplementary material for: An Application of the Social Cognitive Career Theory Model of Career Self-Management to College Athletes’ Career Planning for Life After Sport
Source: Front Psychol. 2020 Jan 24;11:9. doi: 10.3389/fpsyg.2020.00009 (PMC6993061; doi:10.3389/fpsyg.2020.00009)
Supplement: Supplementary file 1 [file Data_Sheet_1.docx]

**Appendix A**

**Measurement scales**

| **Construct** | **Scale reference** | **Adapted scale** |
| --- | --- | --- |
| Career Planning | Career Planning subscale of the Student Developmental Task and Lifestyle Inventory by Winston, Miller and Prince (1987): CP3 and CP8  Career Maturity Inventory by Savickas and Porfeli (2011): CP1  New Scale Items: CP2, CP4, CP5, CP6, and CP7 | - CP1*: I’m having difficulties preparing myself for a career after college sport. - CP2: I have gathered detailed information about career requirements, employment trends, and ways of getting into occupations that interest me. - CP3: I have formulated a viable plan for my career after college sport. - CP4: I have a good understanding of the steps I need to take to reach my career goals. - CP5: I am taking the steps necessary to reach my career goals. - CP6*: I am unsure about what my career plans for life after sport should be yet. - CP7*: I am too busy at this point to make career plans for life after college. - CP8: I have determined a specific plan to gain practical experience in the field I plan to pursue after college. |
| Self-Efficacy | Adapted from Career Decision Self-Efficacy-Short Form scale by Betz, Klein, & Taylor (1996) | - SE1: Select career options that best match your interests, values, skills, personality, and lifestyle. - SE2: Find information on the Internet about careers you are interested in. - SE3: Identify employers, companies, and organizations relevant to your career possibilities. - SE4: Talk with professionals in the field(s) you are interested in. - SE5: Gather detailed information about career requirements, employment trends, and ways of getting into occupations that interest you. - SE6: Find information about graduate or professional schools. - SE7: Prepare a good resume. - SE8: Determine the steps necessary to reach your career goals. - SE9: Gain professional experience and skills related to your career goals. - SE10: Make a plan of your career goals for the next five years. - SE11: Successfully manage the job interview process. - SE12: Integrate your knowledge about yourself and job market trends to develop a viable career plan. |
| Career Goals | Career Goal Setting by Mu (1998): CG1  New Scale Items: CG2, CG3, CG4, and CG5) | - CG1: I have a clear set of goals for my career after sport. - CG2: I think that the career goals that I intend to pursue are realistic to attain. - CG3: Achieving my career goals is one of my priorities. - CG4: I know where my life is headed after sport. - CG5: In the past year, I have discussed my career goals with professionals working in my field(s) of interest. |
| Perceived Career Planning Barriers | Perceptions of Educational Barriers Scale – Revised (McWhirter, 2000)  New Scale Items: B2, B3, B5, B6, B7, B8, B9, and B10 | - B1: Not being smart enough - B2: Feelings of isolation once I graduate - B3: Lack of career preparation - B4: Not being confident enough - B5: A saturated or difficult job market - B6: Not picking the right academic major - B7: Lack of required professional qualifications - B8: Taking too much time to find a job - B9: Lack of control over my career decisions - B10: Lack of career guidance |
| Perceived Career Planning Support from Coaches | Adapted from Career Influence Inventory by Fisher and Stafford (1999) | My coach(es)…   - CS1: Are willing to help me set realistic career goals and make suitable career plans for life after sport. - CS2: Are interested in my career plans for life after sport. - CS3: Are role model(s) for me. - CS4: Encourage me to pursue my career goals. - CS5: Believe I have the work ethic to follow through with my career plans. - CS6: Believe that I am resilient enough to face and overcome obstacles. - CS7: Make me feel that I can succeed in achieving my career aspirations. |
| Openness | Big Five Inventory by John and Srivastava (1999) | - O1: Is original, comes up with new ideas - O2: Values artistic, aesthetic experiences - O3: Has an active imagination - O4: Is curious about many different things |
| Conscientiousness | Big Five Inventory by John and Srivastava (1999) | - C1: Does a thorough job - C2*: Tends to be lazy - C3: Does things efficiently - C4: Makes plans and follows through with them |
| Extraversion | Big Five Inventory by John and Srivastava (1999) | - E1: Is talkative - E2: Is outgoing, sociable - E3*: Is reserved - E4*: Tends to be quiet |
| Agreeableness | Big Five Inventory by John and Srivastava (1999) | - A1*: Is sometimes rude to others - A2: Has a forgiving nature - A3: Is considerate and kind to almost everyone - A4: Is generally trusting |
| Neuroticism | Big Five Inventory by John and Srivastava (1999) | - N1: Worries a lot - N2*: Remains calm in tense situations - N3*: Is relaxed, handles stress well - N4: Can be moody |

Note: * Reverse-coded item.
